# 3-(3-Acetyl­anilino)-1-ferrocenylpropan-1-one

**DOI:** 10.1107/S1600536812028796

**Published:** 2012-06-30

**Authors:** Sladjana B. Novaković, Dragana Stevanović, Vladimir Divjaković, Goran A. Bogdanović, Rastko D. Vukićević

**Affiliations:** a ’Vinča’ Institute of Nuclear Sciences, Laboratory of Theoretical Physics and Condensed Matter Physics, University of Belgrade, PO Box 522, 11001 Belgrade, Serbia; b Department of Chemistry, Faculty of Science, University of Kragujevac, R. Domanovića 12, 34000 Kragujevac, Serbia; c Department of Physics, Faculty of Sciences, University of Novi Sad, Trg Dositeja Obradovića 4, 21000 Novi Sad, Serbia

## Abstract

The title ferrocene-containing Mannich base, [Fe(C_5_H_5_)(C_16_H_16_NO_2_)], crystallizes with two independent mol­ecules (*A* and *B*) in the asymmetric unit. Mol­ecules *A* and *B* have similar conformations. The dihedral angles between the best planes of the benzene and substituted cyclo­penta­dienyl rings are 88.59 (9) and 84.39 (10)° in *A* and *B*, respectively. In the crystal, the independent mol­ecules form centrosymmetric dimers *via* corresponding N—H⋯O hydrogen bonds. The dimers further arrange *via* C—H⋯π and C—H⋯O inter­actions. There are no significant inter­actions between the *A* and *B* mol­ecules.

## Related literature

For the physico-chemical properties of ferrocene-based compounds, see: Togni & Hayashi (1995 ▶). For related structures and details of the synthesis, see: Damljanović *et al.* (2011 ▶); Pejović *et al.* (2012 ▶); Stevanović *et al.* (2012 ▶); Leka *et al.* (2012*a* ▶,*b* ▶,*c* ▶).
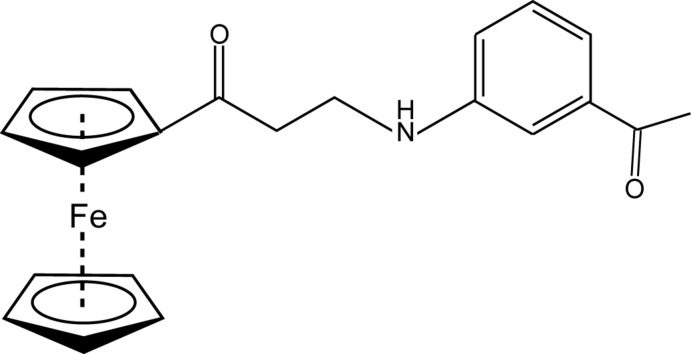


## Experimental

### 

#### Crystal data

[Fe(C_5_H_5_)(C_16_H_16_NO_2_)]
*M* *_r_* = 375.24
Monoclinic, 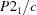

*a* = 22.7768 (8) Å
*b* = 7.3978 (1) Å
*c* = 22.2118 (7) Å
β = 109.642 (4)°
*V* = 3524.87 (19) Å^3^
*Z* = 8
Mo *K*α radiation
μ = 0.87 mm^−1^
*T* = 293 K
0.14 × 0.10 × 0.08 mm

#### Data collection

Oxford Diffraction Xcalibur Sapphire3 Gemini diffractometer
Absorption correction: multi-scan (*CrysAlis PRO*; Oxford Diffraction, 2009 ▶) *T*_min_ = 0.947, *T*_max_ = 1.000
21526 measured reflections
8197 independent reflections
6146 reflections with *I* > 2σ(*I*)
*R*_int_ = 0.029

#### Refinement

*R*[*F* ^2^ > 2σ(*F* ^2^)] = 0.057
*wR*(*F* ^2^) = 0.112
*S* = 1.13
8197 reflections
461 parameters
H atoms treated by a mixture of independent and constrained refinement
Δρ_max_ = 0.29 e Å^−3^
Δρ_min_ = −0.36 e Å^−3^

### 

Data collection: *CrysAlis PRO* (Oxford Diffraction, 2009 ▶); cell refinement: *CrysAlis PRO*; data reduction: *CrysAlis PRO*; program(s) used to solve structure: *SHELXS97* (Sheldrick, 2008 ▶); program(s) used to refine structure: *SHELXL97* (Sheldrick, 2008 ▶); molecular graphics: *ORTEP-3* (Farrugia, 1997 ▶) and *Mercury* (Macrae *et al.*, 2006 ▶); software used to prepare material for publication: *WinGX* (Farrugia, 1999 ▶), *PLATON* (Spek, 2009 ▶) and *PARST* (Nardelli, 1995 ▶).

## Supplementary Material

Crystal structure: contains datablock(s) I, global. DOI: 10.1107/S1600536812028796/bt5949sup1.cif

Structure factors: contains datablock(s) I. DOI: 10.1107/S1600536812028796/bt5949Isup2.hkl

Additional supplementary materials:  crystallographic information; 3D view; checkCIF report

## Figures and Tables

**Table 1:** Hydrogen-bond geometry (Å, °) *Cg*2*A* and *Cg*2*B* are the centroids of the C6*A*–C10*A* and C6*B*–C10*B* rings, respectively.

*D*—H⋯*A*	*D*—H	H⋯*A*	*D*⋯*A*	*D*—H⋯*A*
N1*A*—H1*NA*⋯O1*A* ^i^	0.78 (4)	2.40 (3)	3.162 (4)	166 (3)
N1*B*—H1*NB*⋯O1*B* ^ii^	0.80 (4)	2.46 (3)	3.253 (4)	167 (3)
C9*A*—H9*A*⋯O2*A* ^iii^	0.93	2.49	3.403 (3)	166
C12*A*—H12*A*⋯O1*A* ^iv^	0.97	2.67	3.517 (4)	146
C19*A*—H19*A*⋯O1*A* ^i^	0.93	2.69	3.449 (4)	139
C18*B*—H18*B*⋯O2*B* ^v^	0.93	2.49	3.336 (4)	152
C7*A*—H7*A*⋯*Cg*2A^vi^	0.93	2.98	3.721 (4)	137
C7*B*—H7*B*⋯*Cg*2B^vii^	0.93	2.96	3.781 (5)	148

Symmetry codes: (i) 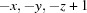; (ii) 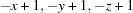; (iii) 
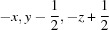
; (iv) 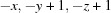; (v) 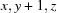; (vi) 
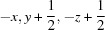
; (vii) 
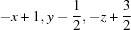
.

**Fig. 1.**
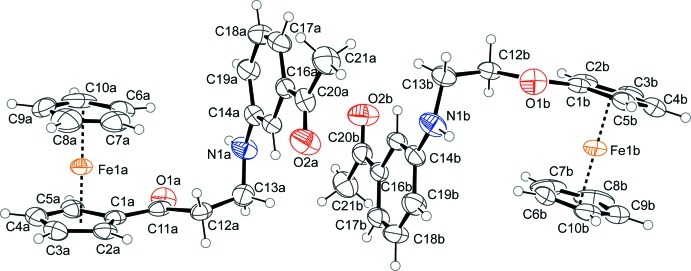
The molecular structure of the title compound with atom labels and 40% probability displacement ellipsoids for non-H atoms.

**Fig. 2.**
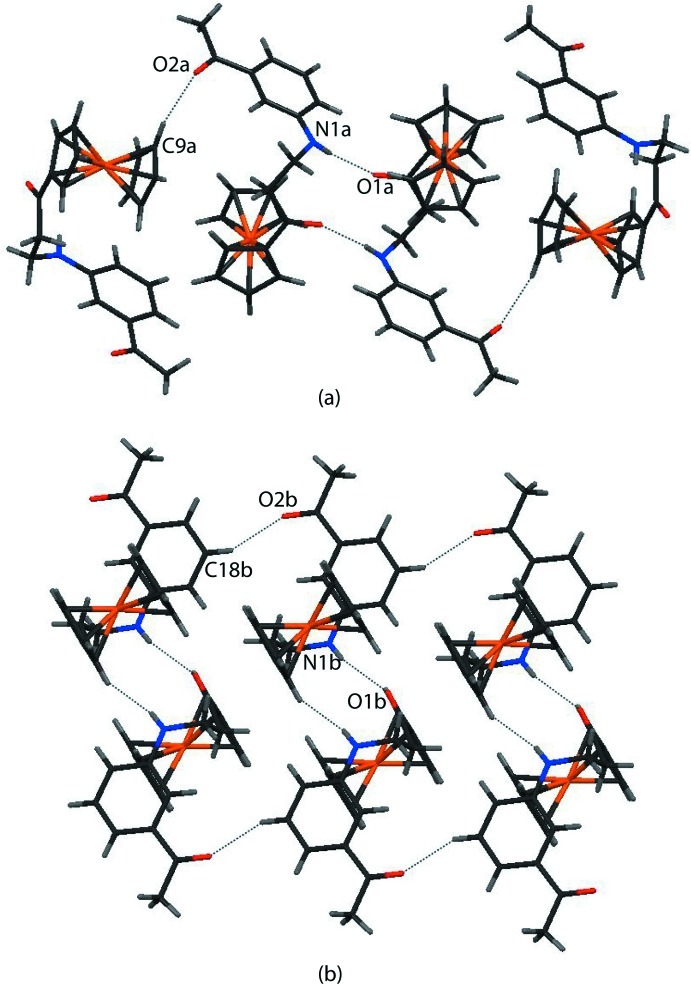
The interconnection of AA dimers (a) and BB dimers (b) into corresponding chains via dissimilar C—H···O interactions.

## supplementary crystallographic information

### Comment

Derivatives of ferrocene have attracted great interest due to their physical, chemical and biological properties (Togni & Hayashi, 1995). In the course of our studies of different ferrocene derivatives containing two or more heteroatoms, we have synthesized and determined the crystal structures of a series of 3-(arylamino)-1-ferrocenylpropan-1-ones (Damljanović *et al.* 2011, Pejović *et al.* 2012, Stevanović *et al.* 2012 Leka *et al.* 2012*a*,*b*,*c*). The present derivative 1-ferrocenyl-3-(3-acetylphenylamino)propan-1-one, crystallizes with two independent molecules (A and B) in the asymmetric unit (Fig. 1). The cyclopentadienyl rings (Cp) within the Fc unit of molecules A and B take a nearly eclipsed geometry; the corresponding torsion angle C1—Cg1—Cg2—C6 has a value of 2.8 and 3.2°, respectively (Cg is centroid of the corresponding Cp ring). Both molecules display a conformation similar to that of previously reported derivatives containing *meta*-substituted phenyl rings.

The torsion angles C1—C11—C12—C13, C11—C12—C13—N1 and C12—C13—N1—C4 within the aliphatic fragment are -172.0 (2)/167.2 (2), 68.4 (3)/-70.4 (3) and 76.0 (4)/-77.0 (4)° (first value corresponds to molecule A, while the second one corresponds to molecule B). Inversion related molecules arrange into AA and BB dimers *via* corresponding N1—H1n···O1 hydrogen bonds. The AA and BB dimers further arrange into separate chains *via* dissimilar C—H···O interactions. In these interactions the acetyl O2 atom engages as an acceptor. On the other hand, the C—H donors engaged in these interactions are not equivalent as the A molecules use cyclopentadienyl while B molecules use phenyl fragments (Fig. 2). The molecules of the same type also interact by relatively strong C—H···π interaction which in both cases include the unsubstituted Cp ring, C7a— H7a···Cg2a^i^: H···Cg 2.98 Å, H—Perp 2.91 Å, *X*—H···Cg 137°, (i = -*x*, *y* + 1/2, -*z* + 1/2) and C7b—H7b···Cg2 b^ii^ H···Cg 2.96 Å H—Perp 2.72 Å, *X*—H···Cg 148 °, (ii = -*x* + 1, *y* - 1/2, -*z* + 3/2). There are no significant interactions between the A and B molecules.

### Experimental

An aza-Michael addition of arylamines to a conjugated enone, acryloylferrocene, has been achieved by ultrasonic irradiation of the mixture of these reactants and the catalyst - montmorillonite K-10. This solvent-free reaction, yielding ferrocene containing Mannich bases (3-(arylamino)-1-ferrocenylpropan-1-ones), has been performed through the use of a simple ultrasonic cleaner. The details of the synthesis are described by Pejović *et al.* (2012*b*).

### Refinement

H atoms bonded to C atoms were placed at geometrically calculated positions and refined using a riding model. C—H distances were fixed to 0.93, 0.97 and 0.96 Å for aromatic, methylene and methyl C atoms, respectively. The *U*_iso_(H) values were set to 1.2 times *U*_eq_ of the corresponding aromatic and methylene C atoms. The *U*_eq_ values of the H atoms attached to methyl C atoms were set equal to 1.5 times *U*_eq_ of the parent atom. H atoms attached to N atoms were refined isotropically.

### Crystal data

**Table:**

[Fe(C_5_H_5_)(C_16_H_16_NO_2_)]	*F*(000) = 1568
*M**_r_* = 375.24	*D*_x_ = 1.414 Mg m^−^^3^
Monoclinic, *P*2_1_/*c*	Mo *K*α radiation, λ = 0.71073 Å
Hall symbol: -P 2ybc	Cell parameters from 7645 reflections
*a* = 22.7768 (8) Å	θ = 3.0–29.0°
*b* = 7.3978 (1) Å	µ = 0.87 mm^−^^1^
*c* = 22.2118 (7) Å	*T* = 293 K
β = 109.642 (4)°	Prismatic, orange
*V* = 3524.87 (19) Å^3^	0.14 × 0.10 × 0.08 mm
*Z* = 8	

### Data collection

**Table:**

Oxford Diffraction Xcalibur, Sapphire3, Gemini diffractometer	8197 independent reflections
Radiation source: Enhance (Mo) X-ray Source	6146 reflections with *I* > 2σ(*I*)
Graphite monochromator	*R*_int_ = 0.029
Detector resolution: 16.3280 pixels mm^-1^	θ_max_ = 29.0°, θ_min_ = 3.0°
ω scans	*h* = −29→17
Absorption correction: multi-scan (*CrysAlis PRO*; Oxford Diffraction, 2009)	*k* = −10→10
*T*_min_ = 0.947, *T*_max_ = 1.000	*l* = −28→29
21526 measured reflections	

### Refinement

**Table:**

Refinement on *F*^2^	Primary atom site location: structure-invariant direct methods
Least-squares matrix: full	Secondary atom site location: difference Fourier map
*R*[*F*^2^ > 2σ(*F*^2^)] = 0.057	Hydrogen site location: inferred from neighbouring sites
*wR*(*F*^2^) = 0.112	H atoms treated by a mixture of independent and constrained refinement
*S* = 1.13	*w* = 1/[σ^2^(*F*_o_^2^) + (0.0329*P*)^2^ + 1.5937*P*] where *P* = (*F*_o_^2^ + 2*F*_c_^2^)/3
8197 reflections	(Δ/σ)_max_ = 0.001
461 parameters	Δρ_max_ = 0.29 e Å^−^^3^
0 restraints	Δρ_min_ = −0.36 e Å^−^^3^

### Special details

**Table:**

Experimental. Empirical absorption correction using spherical harmonics, implemented in SCALE3 ABSPACK scaling algorithm. 'CrysAlisPro, (Oxford Diffraction, 2009)'

### Fractional atomic coordinates and isotropic or equivalent isotropic displacement parameters (Å^2^)

**Table:**

	*x*	*y*	*z*	*U*_iso_*/*U*_eq_	
Fe1A	−0.081087 (18)	0.18406 (5)	0.305745 (18)	0.04314 (12)	
O1A	−0.02446 (10)	0.2106 (3)	0.48588 (10)	0.0623 (6)	
O2A	0.23814 (11)	0.3642 (3)	0.37407 (12)	0.0775 (7)	
N1A	0.11138 (14)	0.1132 (4)	0.50418 (14)	0.0629 (8)	
C1A	−0.06883 (12)	0.3385 (3)	0.38385 (13)	0.0423 (6)	
C2A	−0.06635 (14)	0.4463 (3)	0.33120 (14)	0.0486 (7)	
H2A	−0.0325	0.5151	0.3302	0.058*	
C3A	−0.12352 (15)	0.4306 (4)	0.28136 (16)	0.0597 (8)	
H3A	−0.1343	0.4872	0.2417	0.072*	
C4A	−0.16201 (15)	0.3133 (4)	0.30205 (17)	0.0621 (8)	
H4A	−0.2025	0.2795	0.2781	0.074*	
C5A	−0.12901 (13)	0.2562 (4)	0.36479 (15)	0.0532 (7)	
H5A	−0.1438	0.1786	0.3894	0.064*	
C6A	−0.01325 (17)	−0.0078 (4)	0.32782 (16)	0.0658 (9)	
H6A	0.0170	−0.0251	0.3677	0.079*	
C7A	−0.00816 (17)	0.1032 (5)	0.27946 (19)	0.0699 (10)	
H7A	0.0263	0.1732	0.2814	0.084*	
C8A	−0.0624 (2)	0.0925 (6)	0.22836 (18)	0.0834 (12)	
H8A	−0.0709	0.1544	0.1899	0.100*	
C9A	−0.10267 (18)	−0.0259 (6)	0.2435 (2)	0.0933 (15)	
H9A	−0.1426	−0.0578	0.2172	0.112*	
C10A	−0.0718 (2)	−0.0885 (4)	0.3058 (2)	0.0819 (13)	
H10A	−0.0877	−0.1696	0.3283	0.098*	
C11A	−0.01706 (13)	0.2991 (3)	0.44233 (13)	0.0455 (6)	
C12A	0.04656 (13)	0.3730 (4)	0.44832 (13)	0.0476 (7)	
H12A	0.0455	0.5039	0.4507	0.057*	
H12B	0.0561	0.3414	0.4103	0.057*	
C13A	0.09779 (14)	0.3020 (4)	0.50651 (14)	0.0592 (8)	
H13A	0.1355	0.3703	0.5117	0.071*	
H13B	0.0860	0.3238	0.5440	0.071*	
C14A	0.14484 (13)	0.0446 (4)	0.46782 (13)	0.0479 (7)	
C15A	0.17757 (12)	0.1524 (4)	0.43880 (12)	0.0450 (6)	
H15A	0.1748	0.2775	0.4413	0.054*	
C16A	0.21447 (12)	0.0782 (4)	0.40605 (12)	0.0457 (6)	
C17A	0.21819 (14)	−0.1082 (4)	0.40162 (14)	0.0568 (8)	
H17A	0.2428	−0.1597	0.3802	0.068*	
C18A	0.18474 (15)	−0.2168 (4)	0.42941 (15)	0.0614 (8)	
H18A	0.1867	−0.3418	0.4261	0.074*	
C19A	0.14905 (14)	−0.1431 (4)	0.46150 (14)	0.0554 (7)	
H19A	0.1270	−0.2189	0.4796	0.066*	
C20A	0.24742 (13)	0.2031 (4)	0.37585 (13)	0.0517 (7)	
C21A	0.29297 (16)	0.1271 (5)	0.34724 (17)	0.0735 (10)	
H21A	0.2714	0.0505	0.3118	0.110*	
H21B	0.3240	0.0578	0.3789	0.110*	
H21C	0.3128	0.2241	0.3327	0.110*	
Fe1B	0.584630 (19)	0.44297 (5)	0.692237 (17)	0.04318 (12)	
O1B	0.53611 (10)	0.3022 (3)	0.51869 (9)	0.0588 (5)	
O2B	0.27338 (13)	0.0162 (3)	0.62706 (13)	0.0863 (8)	
N1B	0.39339 (14)	0.3325 (4)	0.50188 (13)	0.0618 (7)	
C1B	0.58556 (13)	0.2491 (3)	0.62822 (12)	0.0415 (6)	
C2B	0.59017 (15)	0.1705 (4)	0.68891 (13)	0.0531 (8)	
H2B	0.5623	0.0889	0.6964	0.064*	
C3B	0.64465 (16)	0.2398 (4)	0.73505 (15)	0.0626 (9)	
H3B	0.6589	0.2115	0.7784	0.075*	
C4B	0.67359 (15)	0.3584 (4)	0.70438 (15)	0.0588 (8)	
H4B	0.7102	0.4221	0.7241	0.071*	
C5B	0.63833 (13)	0.3650 (4)	0.63939 (13)	0.0492 (7)	
H5B	0.6476	0.4333	0.6086	0.059*	
C6B	0.50605 (16)	0.5941 (5)	0.6572 (2)	0.0779 (11)	
H6B	0.4756	0.5806	0.6172	0.093*	
C7B	0.5077 (2)	0.5053 (5)	0.7140 (3)	0.111 (2)	
H7B	0.4791	0.4222	0.7192	0.134*	
C8B	0.5628 (3)	0.5707 (6)	0.76194 (19)	0.0965 (16)	
H8B	0.5769	0.5373	0.8048	0.116*	
C9B	0.59092 (17)	0.6895 (4)	0.73422 (18)	0.0696 (10)	
H9B	0.6276	0.7518	0.7550	0.084*	
C10B	0.55702 (16)	0.7029 (4)	0.67149 (17)	0.0609 (8)	
H10B	0.5671	0.7760	0.6423	0.073*	
C11B	0.53347 (13)	0.2336 (3)	0.56780 (12)	0.0427 (6)	
C12B	0.47645 (13)	0.1280 (4)	0.56710 (13)	0.0470 (7)	
H12C	0.4868	0.0004	0.5715	0.056*	
H12D	0.4650	0.1635	0.6037	0.056*	
C13B	0.42102 (14)	0.1555 (4)	0.50708 (13)	0.0547 (8)	
H13C	0.4340	0.1353	0.4703	0.066*	
H13D	0.3896	0.0657	0.5059	0.066*	
C14B	0.35504 (13)	0.3833 (4)	0.53600 (12)	0.0466 (7)	
C15B	0.32807 (12)	0.2599 (4)	0.56590 (12)	0.0462 (7)	
H15B	0.3371	0.1376	0.5648	0.055*	
C16B	0.28760 (12)	0.3157 (4)	0.59755 (12)	0.0458 (6)	
C17B	0.27401 (14)	0.4969 (4)	0.59914 (14)	0.0583 (8)	
H17B	0.2470	0.5357	0.6200	0.070*	
C18B	0.30080 (16)	0.6205 (4)	0.56956 (15)	0.0651 (9)	
H18B	0.2918	0.7427	0.5708	0.078*	
C19B	0.34035 (15)	0.5658 (4)	0.53854 (15)	0.0606 (8)	
H19B	0.3577	0.6514	0.5189	0.073*	
C20B	0.26079 (14)	0.1741 (5)	0.62884 (14)	0.0570 (8)	
C21B	0.21885 (16)	0.2309 (5)	0.66479 (16)	0.0763 (10)	
H21D	0.2039	0.1258	0.6805	0.114*	
H21E	0.1841	0.2973	0.6368	0.114*	
H21F	0.2416	0.3062	0.7001	0.114*	
H1NB	0.4136 (15)	0.412 (4)	0.4938 (15)	0.067 (12)*	
H1NA	0.0876 (16)	0.046 (5)	0.5099 (16)	0.069 (12)*	

### Atomic displacement parameters (Å^2^)

**Table:**

	*U*^11^	*U*^22^	*U*^33^	*U*^12^	*U*^13^	*U*^23^
Fe1A	0.0454 (2)	0.0316 (2)	0.0589 (2)	0.00040 (17)	0.02608 (19)	−0.00628 (17)
O1A	0.0670 (14)	0.0692 (14)	0.0641 (13)	−0.0013 (11)	0.0396 (11)	0.0046 (11)
O2A	0.0756 (17)	0.0597 (15)	0.1129 (19)	0.0065 (13)	0.0524 (14)	0.0192 (14)
N1A	0.0641 (19)	0.0639 (18)	0.0759 (18)	0.0081 (15)	0.0438 (15)	0.0074 (15)
C1A	0.0450 (16)	0.0299 (13)	0.0610 (17)	0.0024 (11)	0.0298 (13)	−0.0096 (12)
C2A	0.0534 (18)	0.0280 (13)	0.0715 (18)	0.0014 (12)	0.0303 (15)	0.0003 (13)
C3A	0.060 (2)	0.0422 (17)	0.073 (2)	0.0126 (15)	0.0184 (16)	0.0042 (15)
C4A	0.0450 (18)	0.0497 (18)	0.091 (2)	0.0066 (15)	0.0228 (17)	−0.0142 (17)
C5A	0.0469 (17)	0.0449 (16)	0.080 (2)	0.0004 (13)	0.0380 (16)	−0.0093 (15)
C6A	0.067 (2)	0.0545 (19)	0.074 (2)	0.0249 (18)	0.0217 (18)	−0.0140 (17)
C7A	0.070 (2)	0.059 (2)	0.103 (3)	−0.0033 (18)	0.058 (2)	−0.020 (2)
C8A	0.105 (3)	0.087 (3)	0.067 (2)	0.023 (3)	0.040 (2)	−0.015 (2)
C9A	0.059 (2)	0.081 (3)	0.129 (4)	0.002 (2)	0.017 (2)	−0.068 (3)
C10A	0.104 (3)	0.0265 (16)	0.147 (4)	0.0020 (18)	0.084 (3)	−0.013 (2)
C11A	0.0554 (17)	0.0337 (14)	0.0595 (17)	−0.0009 (13)	0.0355 (14)	−0.0115 (13)
C12A	0.0508 (17)	0.0402 (15)	0.0593 (17)	−0.0036 (13)	0.0283 (14)	−0.0121 (13)
C13A	0.0544 (19)	0.070 (2)	0.0595 (18)	−0.0013 (16)	0.0279 (15)	−0.0161 (16)
C14A	0.0415 (16)	0.0521 (17)	0.0500 (15)	0.0049 (13)	0.0153 (12)	0.0026 (13)
C15A	0.0404 (15)	0.0420 (15)	0.0521 (16)	0.0053 (12)	0.0147 (12)	−0.0007 (12)
C16A	0.0393 (15)	0.0503 (17)	0.0451 (15)	0.0043 (13)	0.0112 (12)	−0.0005 (13)
C17A	0.058 (2)	0.0585 (19)	0.0576 (18)	0.0153 (16)	0.0246 (15)	−0.0003 (15)
C18A	0.071 (2)	0.0426 (17)	0.072 (2)	0.0066 (15)	0.0260 (17)	0.0025 (15)
C19A	0.0573 (19)	0.0503 (18)	0.0631 (18)	0.0006 (15)	0.0262 (15)	0.0071 (14)
C20A	0.0394 (16)	0.061 (2)	0.0536 (17)	0.0037 (14)	0.0143 (13)	0.0045 (15)
C21A	0.065 (2)	0.085 (2)	0.086 (2)	−0.0001 (19)	0.0458 (19)	0.000 (2)
Fe1B	0.0552 (3)	0.0314 (2)	0.0494 (2)	0.01005 (17)	0.02617 (19)	0.00179 (16)
O1B	0.0706 (14)	0.0640 (13)	0.0508 (11)	−0.0013 (11)	0.0322 (10)	0.0074 (10)
O2B	0.108 (2)	0.0609 (15)	0.114 (2)	0.0019 (15)	0.0685 (17)	−0.0018 (14)
N1B	0.0643 (19)	0.0634 (19)	0.0683 (17)	0.0170 (15)	0.0365 (14)	0.0158 (14)
C1B	0.0535 (17)	0.0294 (13)	0.0509 (15)	0.0117 (12)	0.0297 (13)	0.0010 (11)
C2B	0.075 (2)	0.0303 (14)	0.0614 (18)	0.0166 (14)	0.0330 (16)	0.0084 (13)
C3B	0.080 (2)	0.0477 (18)	0.0513 (17)	0.0283 (17)	0.0113 (16)	0.0068 (14)
C4B	0.0520 (19)	0.0482 (17)	0.073 (2)	0.0169 (15)	0.0174 (16)	−0.0005 (16)
C5B	0.0515 (17)	0.0440 (16)	0.0606 (18)	0.0114 (13)	0.0300 (14)	0.0011 (13)
C6B	0.0431 (19)	0.068 (2)	0.111 (3)	0.0168 (18)	0.0103 (19)	−0.039 (2)
C7B	0.123 (4)	0.0350 (18)	0.244 (6)	−0.012 (2)	0.152 (4)	−0.026 (3)
C8B	0.173 (5)	0.069 (3)	0.079 (3)	0.048 (3)	0.084 (3)	0.009 (2)
C9B	0.067 (2)	0.053 (2)	0.085 (3)	0.0127 (17)	0.0195 (19)	−0.0221 (18)
C10B	0.073 (2)	0.0359 (16)	0.081 (2)	0.0176 (16)	0.0355 (18)	0.0045 (16)
C11B	0.0549 (17)	0.0315 (13)	0.0518 (16)	0.0119 (12)	0.0312 (13)	0.0015 (12)
C12B	0.0552 (18)	0.0378 (14)	0.0568 (16)	0.0083 (13)	0.0307 (14)	0.0018 (13)
C13B	0.0579 (19)	0.0591 (19)	0.0567 (17)	0.0085 (15)	0.0318 (15)	−0.0066 (14)
C14B	0.0431 (16)	0.0531 (17)	0.0415 (14)	0.0116 (13)	0.0113 (12)	0.0025 (13)
C15B	0.0449 (16)	0.0459 (16)	0.0465 (15)	0.0111 (13)	0.0135 (12)	−0.0030 (12)
C16B	0.0385 (15)	0.0538 (17)	0.0424 (14)	0.0069 (13)	0.0100 (11)	−0.0091 (13)
C17B	0.0517 (19)	0.062 (2)	0.0617 (19)	0.0115 (16)	0.0203 (15)	−0.0132 (16)
C18B	0.070 (2)	0.0454 (17)	0.076 (2)	0.0168 (16)	0.0198 (18)	−0.0056 (16)
C19B	0.061 (2)	0.0530 (19)	0.0670 (19)	0.0085 (16)	0.0207 (16)	0.0079 (16)
C20B	0.0475 (18)	0.070 (2)	0.0546 (17)	0.0027 (16)	0.0184 (14)	−0.0096 (16)
C21B	0.068 (2)	0.100 (3)	0.074 (2)	0.010 (2)	0.0410 (18)	0.001 (2)

### Geometric parameters (Å, º)

**Table:**

Fe1A—C1A	2.017 (2)	Fe1B—C8B	2.014 (3)
Fe1A—C2A	2.017 (3)	Fe1B—C7B	2.022 (3)
Fe1A—C8A	2.021 (3)	Fe1B—C2B	2.022 (3)
Fe1A—C7A	2.027 (3)	Fe1B—C1B	2.025 (2)
Fe1A—C10A	2.027 (3)	Fe1B—C10B	2.028 (3)
Fe1A—C9A	2.028 (3)	Fe1B—C6B	2.031 (3)
Fe1A—C6A	2.033 (3)	Fe1B—C9B	2.031 (3)
Fe1A—C5A	2.039 (3)	Fe1B—C3B	2.039 (3)
Fe1A—C3A	2.050 (3)	Fe1B—C5B	2.043 (3)
Fe1A—C4A	2.053 (3)	Fe1B—C4B	2.050 (3)
O1A—C11A	1.226 (3)	O1B—C11B	1.223 (3)
O2A—C20A	1.209 (3)	O2B—C20B	1.206 (4)
N1A—C14A	1.380 (4)	N1B—C14B	1.387 (4)
N1A—C13A	1.435 (4)	N1B—C13B	1.441 (4)
N1A—H1NA	0.78 (3)	N1B—H1NB	0.80 (3)
C1A—C5A	1.428 (4)	C1B—C5B	1.429 (4)
C1A—C2A	1.432 (4)	C1B—C2B	1.439 (3)
C1A—C11A	1.460 (4)	C1B—C11B	1.467 (4)
C2A—C3A	1.402 (4)	C2B—C3B	1.413 (4)
C2A—H2A	0.9300	C2B—H2B	0.9300
C3A—C4A	1.416 (4)	C3B—C4B	1.404 (4)
C3A—H3A	0.9300	C3B—H3B	0.9300
C4A—C5A	1.408 (4)	C4B—C5B	1.397 (4)
C4A—H4A	0.9300	C4B—H4B	0.9300
C5A—H5A	0.9300	C5B—H5B	0.9300
C6A—C7A	1.388 (5)	C6B—C10B	1.360 (5)
C6A—C10A	1.392 (5)	C6B—C7B	1.413 (6)
C6A—H6A	0.9300	C6B—H6B	0.9300
C7A—C8A	1.370 (5)	C7B—C8B	1.430 (6)
C7A—H7A	0.9300	C7B—H7B	0.9300
C8A—C9A	1.389 (6)	C8B—C9B	1.352 (5)
C8A—H8A	0.9300	C8B—H8B	0.9300
C9A—C10A	1.403 (5)	C9B—C10B	1.351 (5)
C9A—H9A	0.9300	C9B—H9B	0.9300
C10A—H10A	0.9300	C10B—H10B	0.9300
C11A—C12A	1.512 (4)	C11B—C12B	1.511 (4)
C12A—C13A	1.515 (4)	C12B—C13B	1.510 (4)
C12A—H12A	0.9700	C12B—H12C	0.9700
C12A—H12B	0.9700	C12B—H12D	0.9700
C13A—H13A	0.9700	C13B—H13C	0.9700
C13A—H13B	0.9700	C13B—H13D	0.9700
C14A—C15A	1.389 (4)	C14B—C15B	1.388 (4)
C14A—C19A	1.402 (4)	C14B—C19B	1.397 (4)
C15A—C16A	1.396 (4)	C15B—C16B	1.396 (3)
C15A—H15A	0.9300	C15B—H15B	0.9300
C16A—C17A	1.387 (4)	C16B—C17B	1.379 (4)
C16A—C20A	1.485 (4)	C16B—C20B	1.497 (4)
C17A—C18A	1.387 (4)	C17B—C18B	1.382 (4)
C17A—H17A	0.9300	C17B—H17B	0.9300
C18A—C19A	1.362 (4)	C18B—C19B	1.366 (4)
C18A—H18A	0.9300	C18B—H18B	0.9300
C19A—H19A	0.9300	C19B—H19B	0.9300
C20A—C21A	1.497 (4)	C20B—C21B	1.497 (4)
C21A—H21A	0.9600	C21B—H21D	0.9600
C21A—H21B	0.9600	C21B—H21E	0.9600
C21A—H21C	0.9600	C21B—H21F	0.9600
			
C1A—Fe1A—C2A	41.59 (10)	C8B—Fe1B—C7B	41.51 (18)
C1A—Fe1A—C8A	155.99 (16)	C8B—Fe1B—C2B	122.03 (15)
C2A—Fe1A—C8A	119.86 (16)	C7B—Fe1B—C2B	107.80 (14)
C1A—Fe1A—C7A	121.67 (13)	C8B—Fe1B—C1B	159.39 (18)
C2A—Fe1A—C7A	106.81 (13)	C7B—Fe1B—C1B	122.96 (17)
C8A—Fe1A—C7A	39.57 (14)	C2B—Fe1B—C1B	41.66 (10)
C1A—Fe1A—C10A	125.37 (16)	C8B—Fe1B—C10B	65.91 (14)
C2A—Fe1A—C10A	161.63 (17)	C7B—Fe1B—C10B	67.09 (14)
C8A—Fe1A—C10A	67.38 (16)	C2B—Fe1B—C10B	162.81 (14)
C7A—Fe1A—C10A	67.14 (14)	C1B—Fe1B—C10B	126.00 (12)
C1A—Fe1A—C9A	162.29 (19)	C8B—Fe1B—C6B	67.84 (17)
C2A—Fe1A—C9A	155.16 (19)	C7B—Fe1B—C6B	40.80 (17)
C8A—Fe1A—C9A	40.11 (16)	C2B—Fe1B—C6B	126.34 (14)
C7A—Fe1A—C9A	67.15 (15)	C1B—Fe1B—C6B	109.68 (12)
C10A—Fe1A—C9A	40.49 (16)	C10B—Fe1B—C6B	39.15 (13)
C1A—Fe1A—C6A	108.29 (12)	C8B—Fe1B—C9B	39.04 (15)
C2A—Fe1A—C6A	124.23 (13)	C7B—Fe1B—C9B	67.59 (15)
C8A—Fe1A—C6A	67.11 (15)	C2B—Fe1B—C9B	156.34 (14)
C7A—Fe1A—C6A	39.97 (13)	C1B—Fe1B—C9B	160.48 (14)
C10A—Fe1A—C6A	40.10 (14)	C10B—Fe1B—C9B	38.87 (13)
C9A—Fe1A—C6A	67.67 (15)	C6B—Fe1B—C9B	66.34 (13)
C1A—Fe1A—C5A	41.22 (10)	C8B—Fe1B—C3B	106.71 (15)
C2A—Fe1A—C5A	69.07 (11)	C7B—Fe1B—C3B	123.75 (19)
C8A—Fe1A—C5A	161.03 (16)	C2B—Fe1B—C3B	40.72 (12)
C7A—Fe1A—C5A	158.43 (15)	C1B—Fe1B—C3B	69.00 (11)
C10A—Fe1A—C5A	109.76 (14)	C10B—Fe1B—C3B	155.95 (15)
C9A—Fe1A—C5A	125.43 (16)	C6B—Fe1B—C3B	161.99 (17)
C6A—Fe1A—C5A	123.73 (14)	C9B—Fe1B—C3B	120.99 (14)
C1A—Fe1A—C3A	68.90 (12)	C8B—Fe1B—C5B	157.6 (2)
C2A—Fe1A—C3A	40.32 (12)	C7B—Fe1B—C5B	159.5 (2)
C8A—Fe1A—C3A	106.78 (15)	C2B—Fe1B—C5B	68.95 (12)
C7A—Fe1A—C3A	123.06 (14)	C1B—Fe1B—C5B	41.12 (11)
C10A—Fe1A—C3A	157.63 (17)	C10B—Fe1B—C5B	109.70 (12)
C9A—Fe1A—C3A	121.11 (17)	C6B—Fe1B—C5B	123.78 (15)
C6A—Fe1A—C3A	159.64 (15)	C9B—Fe1B—C5B	123.63 (14)
C5A—Fe1A—C3A	68.32 (13)	C3B—Fe1B—C5B	67.84 (12)
C1A—Fe1A—C4A	68.50 (12)	C8B—Fe1B—C4B	122.05 (18)
C2A—Fe1A—C4A	68.03 (12)	C7B—Fe1B—C4B	159.4 (2)
C8A—Fe1A—C4A	124.38 (16)	C2B—Fe1B—C4B	68.25 (13)
C7A—Fe1A—C4A	159.59 (15)	C1B—Fe1B—C4B	68.46 (12)
C10A—Fe1A—C4A	123.70 (15)	C10B—Fe1B—C4B	122.47 (14)
C9A—Fe1A—C4A	108.72 (14)	C6B—Fe1B—C4B	157.43 (17)
C6A—Fe1A—C4A	159.05 (15)	C9B—Fe1B—C4B	107.43 (14)
C5A—Fe1A—C4A	40.23 (12)	C3B—Fe1B—C4B	40.15 (12)
C3A—Fe1A—C4A	40.36 (12)	C5B—Fe1B—C4B	39.92 (11)
C14A—N1A—C13A	123.2 (3)	C14B—N1B—C13B	122.7 (3)
C14A—N1A—H1NA	114 (3)	C14B—N1B—H1NB	116 (2)
C13A—N1A—H1NA	117 (3)	C13B—N1B—H1NB	114 (2)
C5A—C1A—C2A	107.0 (2)	C5B—C1B—C2B	106.7 (2)
C5A—C1A—C11A	125.9 (3)	C5B—C1B—C11B	125.5 (2)
C2A—C1A—C11A	126.6 (2)	C2B—C1B—C11B	127.4 (3)
C5A—C1A—Fe1A	70.23 (15)	C5B—C1B—Fe1B	70.11 (15)
C2A—C1A—Fe1A	69.22 (14)	C2B—C1B—Fe1B	69.09 (14)
C11A—C1A—Fe1A	119.28 (17)	C11B—C1B—Fe1B	120.10 (17)
C3A—C2A—C1A	108.5 (3)	C3B—C2B—C1B	107.6 (3)
C3A—C2A—Fe1A	71.10 (16)	C3B—C2B—Fe1B	70.28 (16)
C1A—C2A—Fe1A	69.19 (14)	C1B—C2B—Fe1B	69.25 (14)
C3A—C2A—H2A	125.7	C3B—C2B—H2B	126.2
C1A—C2A—H2A	125.7	C1B—C2B—H2B	126.2
Fe1A—C2A—H2A	125.5	Fe1B—C2B—H2B	125.8
C2A—C3A—C4A	107.8 (3)	C4B—C3B—C2B	108.4 (3)
C2A—C3A—Fe1A	68.58 (15)	C4B—C3B—Fe1B	70.33 (16)
C4A—C3A—Fe1A	69.95 (17)	C2B—C3B—Fe1B	69.00 (16)
C2A—C3A—H3A	126.1	C4B—C3B—H3B	125.8
C4A—C3A—H3A	126.1	C2B—C3B—H3B	125.8
Fe1A—C3A—H3A	127.0	Fe1B—C3B—H3B	126.5
C5A—C4A—C3A	108.8 (3)	C5B—C4B—C3B	108.8 (3)
C5A—C4A—Fe1A	69.34 (16)	C5B—C4B—Fe1B	69.76 (16)
C3A—C4A—Fe1A	69.69 (17)	C3B—C4B—Fe1B	69.52 (18)
C5A—C4A—H4A	125.6	C5B—C4B—H4B	125.6
C3A—C4A—H4A	125.6	C3B—C4B—H4B	125.6
Fe1A—C4A—H4A	127.0	Fe1B—C4B—H4B	126.7
C4A—C5A—C1A	107.8 (3)	C4B—C5B—C1B	108.4 (3)
C4A—C5A—Fe1A	70.43 (17)	C4B—C5B—Fe1B	70.32 (16)
C1A—C5A—Fe1A	68.54 (14)	C1B—C5B—Fe1B	68.77 (14)
C4A—C5A—H5A	126.1	C4B—C5B—H5B	125.8
C1A—C5A—H5A	126.1	C1B—C5B—H5B	125.8
Fe1A—C5A—H5A	126.5	Fe1B—C5B—H5B	126.7
C7A—C6A—C10A	107.5 (3)	C10B—C6B—C7B	107.6 (3)
C7A—C6A—Fe1A	69.79 (18)	C10B—C6B—Fe1B	70.31 (18)
C10A—C6A—Fe1A	69.74 (18)	C7B—C6B—Fe1B	69.3 (2)
C7A—C6A—H6A	126.2	C10B—C6B—H6B	126.2
C10A—C6A—H6A	126.2	C7B—C6B—H6B	126.2
Fe1A—C6A—H6A	125.8	Fe1B—C6B—H6B	125.8
C8A—C7A—C6A	108.7 (3)	C6B—C7B—C8B	105.1 (3)
C8A—C7A—Fe1A	70.0 (2)	C6B—C7B—Fe1B	69.92 (19)
C6A—C7A—Fe1A	70.24 (17)	C8B—C7B—Fe1B	68.9 (2)
C8A—C7A—H7A	125.7	C6B—C7B—H7B	127.5
C6A—C7A—H7A	125.7	C8B—C7B—H7B	127.5
Fe1A—C7A—H7A	125.7	Fe1B—C7B—H7B	125.3
C7A—C8A—C9A	108.7 (4)	C9B—C8B—C7B	108.2 (4)
C7A—C8A—Fe1A	70.45 (19)	C9B—C8B—Fe1B	71.19 (19)
C9A—C8A—Fe1A	70.2 (2)	C7B—C8B—Fe1B	69.6 (2)
C7A—C8A—H8A	125.6	C9B—C8B—H8B	125.9
C9A—C8A—H8A	125.6	C7B—C8B—H8B	125.9
Fe1A—C8A—H8A	125.3	Fe1B—C8B—H8B	125.0
C8A—C9A—C10A	107.1 (3)	C10B—C9B—C8B	108.9 (4)
C8A—C9A—Fe1A	69.7 (2)	C10B—C9B—Fe1B	70.42 (18)
C10A—C9A—Fe1A	69.75 (19)	C8B—C9B—Fe1B	69.8 (2)
C8A—C9A—H9A	126.4	C10B—C9B—H9B	125.6
C10A—C9A—H9A	126.4	C8B—C9B—H9B	125.6
Fe1A—C9A—H9A	125.7	Fe1B—C9B—H9B	125.8
C6A—C10A—C9A	108.0 (3)	C9B—C10B—C6B	110.2 (3)
C6A—C10A—Fe1A	70.17 (18)	C9B—C10B—Fe1B	70.71 (18)
C9A—C10A—Fe1A	69.76 (19)	C6B—C10B—Fe1B	70.54 (18)
C6A—C10A—H10A	126.0	C9B—C10B—H10B	124.9
C9A—C10A—H10A	126.0	C6B—C10B—H10B	124.9
Fe1A—C10A—H10A	125.6	Fe1B—C10B—H10B	125.4
O1A—C11A—C1A	121.6 (3)	O1B—C11B—C1B	121.1 (3)
O1A—C11A—C12A	120.4 (3)	O1B—C11B—C12B	120.3 (3)
C1A—C11A—C12A	118.0 (2)	C1B—C11B—C12B	118.5 (2)
C11A—C12A—C13A	113.0 (2)	C13B—C12B—C11B	113.6 (2)
C11A—C12A—H12A	109.0	C13B—C12B—H12C	108.9
C13A—C12A—H12A	109.0	C11B—C12B—H12C	108.9
C11A—C12A—H12B	109.0	C13B—C12B—H12D	108.9
C13A—C12A—H12B	109.0	C11B—C12B—H12D	108.9
H12A—C12A—H12B	107.8	H12C—C12B—H12D	107.7
N1A—C13A—C12A	114.9 (3)	N1B—C13B—C12B	114.0 (2)
N1A—C13A—H13A	108.6	N1B—C13B—H13C	108.8
C12A—C13A—H13A	108.6	C12B—C13B—H13C	108.8
N1A—C13A—H13B	108.6	N1B—C13B—H13D	108.8
C12A—C13A—H13B	108.6	C12B—C13B—H13D	108.8
H13A—C13A—H13B	107.5	H13C—C13B—H13D	107.7
N1A—C14A—C15A	123.2 (3)	N1B—C14B—C15B	123.0 (3)
N1A—C14A—C19A	119.5 (3)	N1B—C14B—C19B	119.2 (3)
C15A—C14A—C19A	117.2 (3)	C15B—C14B—C19B	117.8 (3)
C14A—C15A—C16A	121.8 (3)	C14B—C15B—C16B	121.3 (3)
C14A—C15A—H15A	119.1	C14B—C15B—H15B	119.3
C16A—C15A—H15A	119.1	C16B—C15B—H15B	119.3
C17A—C16A—C15A	119.3 (3)	C17B—C16B—C15B	119.5 (3)
C17A—C16A—C20A	122.3 (3)	C17B—C16B—C20B	122.6 (3)
C15A—C16A—C20A	118.4 (3)	C15B—C16B—C20B	117.9 (3)
C16A—C17A—C18A	119.2 (3)	C16B—C17B—C18B	119.5 (3)
C16A—C17A—H17A	120.4	C16B—C17B—H17B	120.3
C18A—C17A—H17A	120.4	C18B—C17B—H17B	120.3
C19A—C18A—C17A	121.0 (3)	C19B—C18B—C17B	121.0 (3)
C19A—C18A—H18A	119.5	C19B—C18B—H18B	119.5
C17A—C18A—H18A	119.5	C17B—C18B—H18B	119.5
C18A—C19A—C14A	121.5 (3)	C18B—C19B—C14B	120.9 (3)
C18A—C19A—H19A	119.3	C18B—C19B—H19B	119.5
C14A—C19A—H19A	119.3	C14B—C19B—H19B	119.5
O2A—C20A—C16A	121.3 (3)	O2B—C20B—C16B	121.6 (3)
O2A—C20A—C21A	119.7 (3)	O2B—C20B—C21B	119.4 (3)
C16A—C20A—C21A	119.1 (3)	C16B—C20B—C21B	119.0 (3)
C20A—C21A—H21A	109.5	C20B—C21B—H21D	109.5
C20A—C21A—H21B	109.5	C20B—C21B—H21E	109.5
H21A—C21A—H21B	109.5	H21D—C21B—H21E	109.5
C20A—C21A—H21C	109.5	C20B—C21B—H21F	109.5
H21A—C21A—H21C	109.5	H21D—C21B—H21F	109.5
H21B—C21A—H21C	109.5	H21E—C21B—H21F	109.5

### Hydrogen-bond geometry (Å, º)

*Cg*2*A* and *Cg*2*B* are the centroids of the C6*A*–C10*A* and C6*B*–C10*B* rings, respectively.

**Table:**

*D*—H···*A*	*D*—H	H···*A*	*D*···*A*	*D*—H···*A*
N1*A*—H1*NA*···O1*A*^i^	0.78 (4)	2.40 (3)	3.162 (4)	166 (3)
N1*B*—H1*NB*···O1*B*^ii^	0.80 (4)	2.46 (3)	3.253 (4)	167 (3)
C9*A*—H9*A*···O2*A*^iii^	0.93	2.49	3.403 (3)	166
C12*A*—H12*A*···O1*A*^iv^	0.97	2.67	3.517 (4)	146
C19*A*—H19*A*···O1*A*^i^	0.93	2.69	3.449 (4)	139
C18*B*—H18*B*···O2*B*^v^	0.93	2.49	3.336 (4)	152
C7*A*—H7*A*···*Cg*2A^vi^	0.93	2.98	3.721 (4)	137
C7*B*—H7*B*···*Cg*2B^vii^	0.93	2.96	3.781 (5)	148

Symmetry codes: (i) −*x*, −*y*, −*z*+1; (ii) −*x*+1, −*y*+1, −*z*+1; (iii) −*x*, *y*−1/2, −*z*+1/2; (iv) −*x*, −*y*+1, −*z*+1; (v) *x*, *y*+1, *z*; (vi) −*x*, *y*+1/2, −*z*+1/2; (vii) −*x*+1, *y*−1/2, −*z*+3/2.
